# Data characterizing the ZMIZ1 molecular phenotype of multiple sclerosis

**DOI:** 10.1016/j.dib.2017.02.040

**Published:** 2017-02-21

**Authors:** N. Fewings, P.N. Gatt, F.C. McKay, G.P. Parnell, S.D. Schibeci, J. Edwards, M.A. Basuki, A. Goldinger, M.J. Fabis-Pedrini, A.G. Kermode, C.P. Manrique, J.L. McCauley, D. Nickles, S.E. Baranzini, T. Burke, S. Vucic, G.J. Stewart, D.R. Booth

**Affiliations:** a Centre for Immunology and Allergy Research, Westmead Institute of Medical Research, University of Sydney, Sydney, New South Wales, Australia; b University of Queensland Diamantina Institute, Translational Research Institute, Australia; c Western Australian Neuroscience Research Institute, University of Western Australia, Nedlands, Western Australia, Australia; d Institute for Immunology and Infectious Diseases, Murdoch University, Murdoch, Western Australia, Australia; e John P. Hussman Institute for Human Genomics and the Dr. John T. Macdonald Foundation Department of Human Genetics, University of Miami, Miller School of Medicine, Miami, FL 33136, USA; f Department of Neurology, University of California San Francisco, USA; g Western Clinical School, University of Sydney, Westmead Hospital, Sydney, New South Wales, Australia; h The Queensland Brain Institute, University of Queensland, Australia

**Keywords:** ZMIZ1, Gene expression, Multiple sclerosis, Molecular phenotype

## Abstract

The data presented in this article are related to the research article entitled “The autoimmune risk gene ZMIZ1 is a vitamin D responsived marker of a molecular phenotype of multiple sclerosis” Fewings et al. (2017) [1]. Here we identify the set of genes correlated with ZMIZ1 in multiple cohorts, provide phenotypic details on those cohorts, and identify the genes negatively correlated with ZMIZ1 and the cells predominantly expressing those genes. We identify the metabolic pathways in which the molecular phenotype genes are over-represented. Finally, we present the flow cytometry gating strategy we have used to identify the immune cells from blood which are producing ZMIZ1 and RPS6.

**Specifications Table**

**Table:** 

Subject area	*Biology*
More specific subject area	*Autoimmune Genetics*
Type of data	*Tables, Figures*
How data was acquired	*Molecular phenotypes are determined from transcriptomic data (RNAseq, Microarray) derived from previously published sources*[2], [3], [4]
	*Molecular pathways were analysed using GeneGo, Metacore.*
	*Flow Cytometry data was acquired using Canto II (BD Biosciences) and Fortessa (BD Biosciences) instruments.*
Data format	*Analyzed*
Experimental factors	*Samples are from whole blood collected in to PAXgene tubes for RNA analysis. The source of samples were healthy controls and Multiple Sclerosis patients not on therapy from Australia and the United States.*
Experimental features	*Gene expression in whole blood was interrogated by transcriptomic and RTPCR procedures to identify genes dysregulated in MS and correlated gene sets (mRNA level). Flow cytometry was used to identify immune cell subsets in which ZMIZ1 and RPS6 were most highly expressed (protein level).*
Data source location	*Cohorts of MS patients and controls are from Sydney and Perth in Australia; and Miami and San Francisco in United States.*
Data accessibility	*UCSF CIS Cohort Transcriptome Source Data is in Repository*GSE41846*(url:*https://www.ncbi.nlm.nih.gov/geo/query/acc.cgi?acc=GSE41846*)* *ANZgene Transcriptome Source Data is in Repository*GSE17048*(url:*https://www.ncbi.nlm.nih.gov/geo/query/acc.cgi?acc=GSE17048*)*

**Value of the data**

1. The detailed list of genes defining the ZMIZ1 molecular phenotype in multiple cohorts is described (Supplementary Table 1).
2. The cohorts used to identify the genes in the ZMIZ1 molecular phenotype (Table 1).
3. The genes negatively correlated with ZMIZ1 expression in whole blood and the immune cells in which they are expressed are identified (Fig. 1).
4. The gene pathways indicated as overrepresented with the genes of the ZMIZ1 molecular phenotype are identified (Fig. 2).
5. The flow cytometry gating strategy used to identify the immune cells most highly expressing ZMIZ1 and RPS6 are diagrammed (Fig. 3).
6. A list of Multiple Sclerosis (MS) risk SNPs tested for association with gene expression in whole blood in the study published in [1] (Table 2).

## 1 Data

**Fig. 1:**
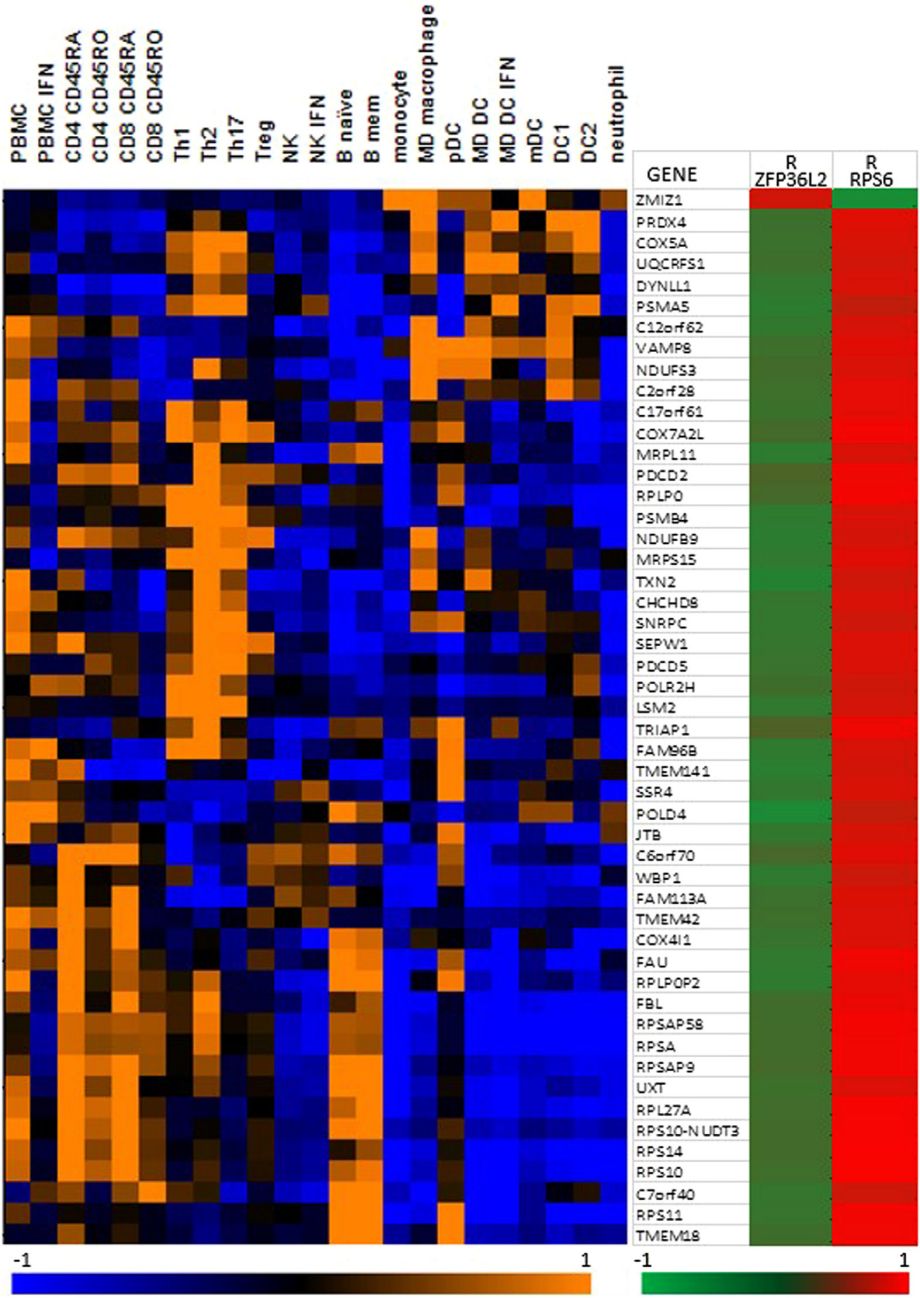
Relative expression in immune cell subsets of ZMIZ1 and the 50 genes whose expression is most highly negatively correlated with ZMIZ1 expression in PAXgene whole blood in multiple sclerosis and healthy controls. (These were determined in the RNASeq cohort: n=32 MS, n=40 healthy controls, [4]. These genes are mostly expressed in lymphocytes. Expression was by RNASeq and colour on heatmap indicates relative expression level: orange is high, blue is low. Cell subsets were ex vivo or in-vitro generated as previously described [7]. Pearson׳s correlation (R) of expression with ZFP36L2 and RPS6 is also shown for each module gene, red is positive correlation and green is negative correlation. ZFP36L2 correlations all less than r=-0.27 (p=0.02), RPS6 correlations all greater than r=0.54 (p=8.9E-07). (For interpretation of the references to color in this figure legend, the reader is referred to the web version of this article.)

**Fig. 2:**
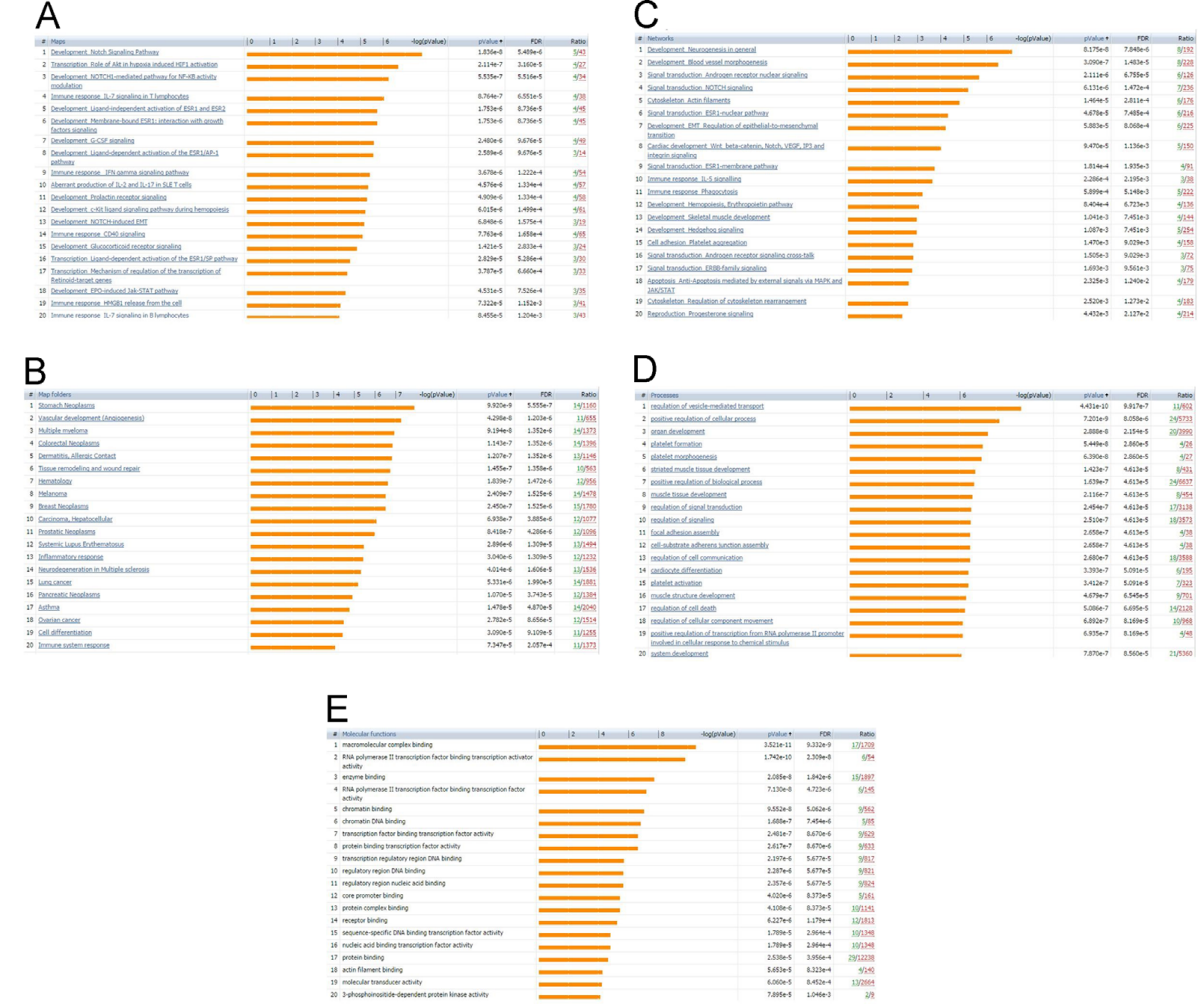
Gene pathways, diseases, process networks, processes, and molecular functions enriched for the ZMIZ1 Molecular Module genes (the 200 genes with expression most highly positively correlated with ZMIZ1 gene expression in whole blood). The 20 most over-represented items in the module are shown for A. Pathway maps; B. Pathway map folders (diseases); C. Process networks; D. Processes; E. Molecular functions. Enrichment analysis using Gene Ontology (GO) software (http://geneontology.org/); with items listed in order of significance; p value shows the probability that the module is not over-represented; FDR: false discovery rate; ratio is the number of genes from the ZMIZ1 module in the pathway, compared to the total number of genes in the pathway.

**Fig. 3:**
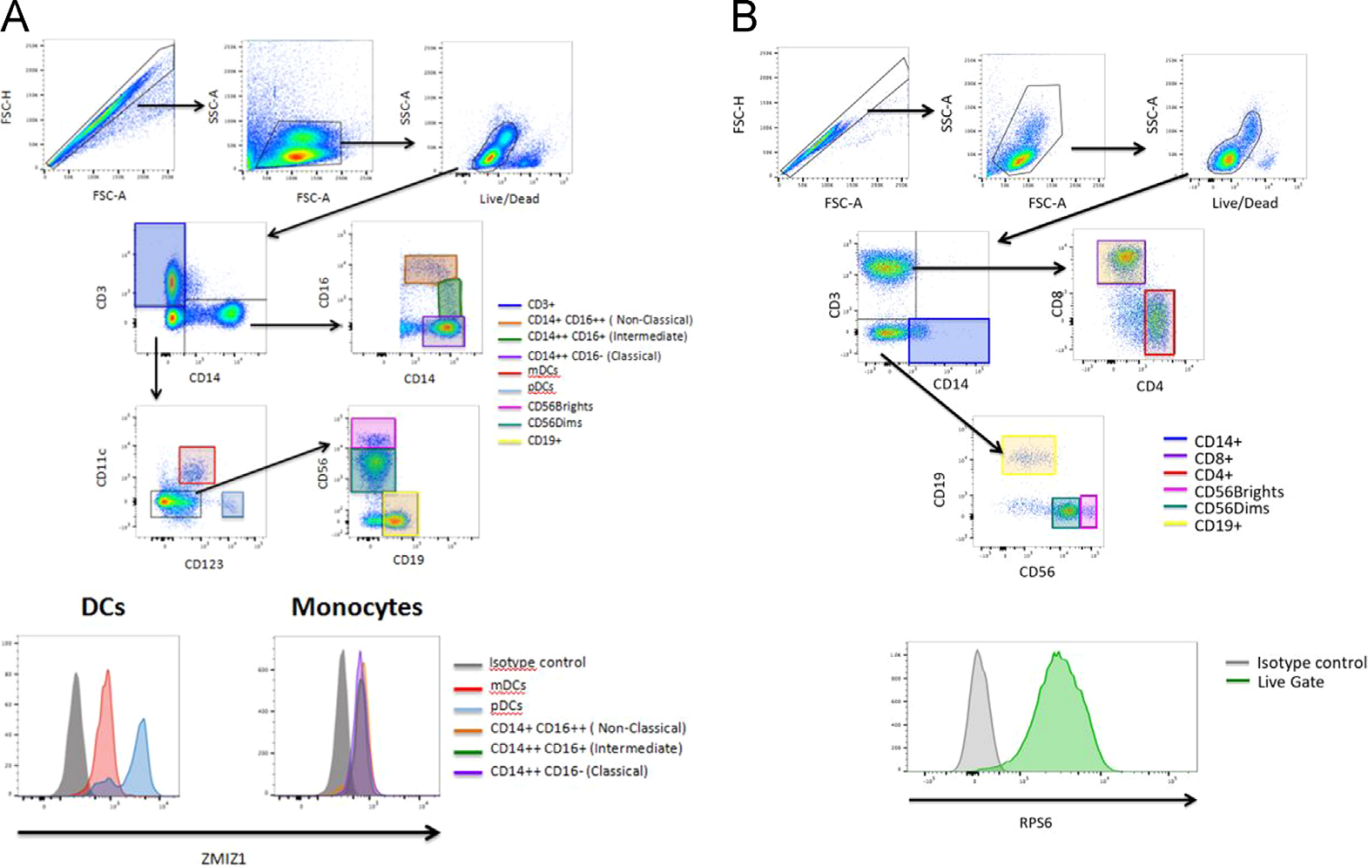
Flow cytometric gating of PBMC subsets for determination of ZMIZ1 and RPS6 protein expression. (A) ZMIZ1 panel: progressive gating of subsets of interest, and ZMIZ1 median fluorescence intensity of DC and monocyte subsets relative to isotype control; (B) RPS6 panel: gating of live PBMCs and their median fluorescence intensity of RPS6 relative to isotype control.

We have recently described a gene, ZMIZ1, whose expression is dysregulated in the blood of people with MS [1]. From transcriptomic data, the expression of this gene is tightly correlated with that of many others. The set of genes whose expression is most correlated with ZMIZ1 across cohorts from Australia and the United States is defined in Supplementary Table 1. The cohort details are described in Table 1. The genes whose expression is most positively correlated with that of ZMIZ1 is previously described [1]. The genes most negatively correlated with ZMIZ1, and the immune cell subsets in which they are predominantly produced, are shown in Fig. 1. Gene pathway analysis was used to identify the types of molecular pathways most overrepresented in the gene lists of the ZMIZ1 molecular phenotype (Fig. 2). Specifically, GeneGo Maps, GeneGo Map folders, GeneGo Networks, Gene Ontology processes, and Gene Ontology molecular functions over-represented are shown in Figs. 2A–E respectively. The flow cytometry gating strategy to identify the immune cell subsets expressing ZMIZ1 and RPS6 is described in Figs. 3A and B respectively. A list of MS risk single nucleotide polymorphisms (SNPs) tested for association with gene expression in whole blood in this study is presented in Table 2.

## 2 Experimental design, materials and methods

### 2.1 Cohorts

Untreated MS patients who had not been on immunomodulatory therapies for at least three months and age-matched healthy controls were recruited for the following cohorts. The molecular phenotypes were determined from previously described transcriptomic cohorts (all PAXgene whole blood samples): ANZgene (2010) [2]; clinically isolated syndrome (CIS; or first clinical diagnosis of central nervous system demyelination) [3] and RNAseq [4], [5]. All MS patients were diagnosed using the revised MacDonald criteria [6]. All blood was collected with informed consent after the nature and possible consequences of the studies were explained from people with MS and healthy controls with approval from Human Research Ethics Committees.

### 2.2 Molecular phenotypes

The genes most correlated with ZMIZ1, ZFP36L2 and RPS6 expression in PAXgene whole blood was determined by Pearson׳s correlation using an RNAseq dataset of MS patients and healthy controls [4]. To visualise relative expression levels across different immune cell populations for the most correlated genes, a heatmap was generated using an RNAseq dataset of ex-vivo and in-vitro differentiated immune cell subsets, as previously described [7]. These genes were assessed for immune cell transcription factor roles and involvement in molecular pathways using GeneGo Metacore. Genes associated with MS were also noted. We then tested correlations in previously described cohorts used for transcriptomic studies: the ANZgene microarray cohort [2], and the CIS cohort [3].

### 2.3 Flow cytometry

Venous blood was collected in EDTA and peripheral blood mononuclear cells (PBMCs) isolated on Ficoll-Paque Plus (VWR International), washed in phosphate-buffered saline (PBS) and cryopreserved in RPMI 1640 Medium (Life Technologies) containing 2 mM glutamine, 10% heat-inactivated fetal bovine serum (FBS, Fisher Biotec), 10% DMSO and 50 units/ml penicillin and 50 µg/ml streptomycin. PBMCs were thawed, washed in RPMI with 2% FBS, and incubated for 30 min in RPMI with 2% FBS, 10 mM HEPES, 1 mM magnesium chloride and 100 units/ml DNase I (Roche). Cells were stained with Live/Dead Aqua viability stain (Molecular Probes) in PBS on ice for 30 min, washed in PBS, and blocked with 33 µg/ml mouse IgG (Life technologies). Antibodies were as described in [1].

## Figures and Tables

**Table 1:** Usage and source of various cohorts in this work.

**Cohort Name**	**Used in this study in:**	**Numbers of individuals**	**Original Reference**
Sydney PCR cohort	Figures 2, 3, 4, 5, 6, 7, 9	n=39 untreated MS n=40 healthy controls	[1]
Miami cohort	Figures 3, 6, 7	n=73 untreated MS n=32 healthy controls	[1]
Treated MS cohort	Figure 10	Total, n=78: n=10, glatiramer acetate n=18, fingolimod n=20, interferon beta n=23, natalizumab n=7, dimethyl fumarate	[8]
ANZgene (microarray) cohort	Figures 1B, 6, 7	n=99 untreated MS; n=45 healthy controls	[2]
Sydney RNASeq cohort	Figures 1, 3, 6, 7, Supplementary Figure. 1 and 2	n=32 untreated MS; n=40 healthy controls	[4]
CIS cohort	Figure 1B	n=42 CIS	[3]

**Table 2:** List of MS risk SNPs tested for association with gene expression in whole blood in this study.

**Gene**	**SNP**	**Reference**
RPS6KB1	rs180515	[9]
ZMIZ1	rs1782645	[10]
ZFP36L2	rs2163226	[9]
HLADRB1	rs2516049	[10]
CYP27B1	rs10877012	[11]
CYP24A1	rs2248359	[10]

## Appendix A Supplementary material

**Supplementary Table 1 legend.** Expanded gene list of the ZMIZ1 module and comparison across multiple cohorts. **A.** Module of 1656 genes with expression correlated with ZMIZ1 (R=0.63 to 0.91, p<5×10^–11^ to 4×10^–29^, (5×10^−5^ to 8×10^–25^ corrected)) and 621 genes correlated with ZFP36L2 ((R=0.63 to 0.84, p<2×10^−9^ to 2×10^–20^, (5×10^−5^ to 5×10^–16^ corrected)) in the RNASeq cohort. Lists of 200 genes whose expression is most highly positively **B.** or negatively **C.** correlated with ZMIZ1 gene expression in whole blood in 3 separate cohorts. Pearson׳s correlation coefficient (R) for each gene is shown for each cohort, and overlap between the cohorts is shown (Venn diagram).

## References

[1] N.L. Fewings, P.N. Gatt, F.C. McKay, G.P. Parnell, S.D. Schibeci, J. Edwards, et al., The autoimmune risk gene ZMIZ1 is a vitamin D responsive marker of a molecular phenotype of multiple sclerosis, J. Autoimmun. (2017), http://dx.doi.org/10.1016/j.jaut.2016.12.006. 10.1016/j.jaut.2016.12.006 28063629

[2] Gandhi K.S., McKay F.C., Cox M., Riveros C., Armstrong N., Heard R.N. (2010). The multiple sclerosis whole blood mRNA transcriptome and genetic associations indicate dysregulation of specific T cell pathways in pathogenesis. Hum. Mol. Genet..

[3] Nickles D., Chen H.P., Li M.M., Khankhanian P., Madireddy L., Caillier S.J. (2013). Blood RNA profiling in a large cohort of multiple sclerosis patients and healthy controls. Hum. Mol. Genet..

[4] Parnell G.P., Gatt P.N., McKay F.C., Schibeci S., Krupa M., Powell J.E. (2014). Ribosomal protein S6 mRNA is a biomarker upregulated in multiple sclerosis, downregulated by interferon treatment, and affected by season. Mult. Scler..

[5] Parnell G.P., Gatt P.N., Krupa M., Nickles D., McKay F.C., Schibeci S.D. (2014). The autoimmune disease-associated transcription factors EOMES and TBX21 are dysregulated in multiple sclerosis and define a molecular subtype of disease. Clin. Immunol..

[6] Polman C.H., Reingold S.C., Banwell B., Clanet M., Cohen J.A., Filippi M. (2011). Diagnostic criteria for multiple sclerosis: 2010 revisions to the McDonald criteria. Ann. Neurol..

[7] Shahijanian F., Parnell G.P., McKay F.C., Gatt P.N., Shojoei M., O׳Connor K.S. (2014). The CYP27B1 variant associated with an increased risk of autoimmune disease is underexpressed in tolerizing dendritic cells. Hum. Mol. Genet..

[8] McKay F.C., Gatt P.N., Fewings N., Parnell G.P., Schibeci S.D., Basuki M.A. (2016). The low EOMES/TBX21 molecular phenotype in multiple sclerosis reflects CD56+ cell dysregulation and is affected by immunomodulatory therapies. Clin. Immunol..

[9] IMSGC, WTCCC2 (2011). Genetic risk and a primary role for cell-mediated immune mechanisms in multiple sclerosis. Nature.

[10] Beecham A.H., IMSGC (2013). Analysis of immune-related loci identifies 48 new susceptibility variants for multiple sclerosis. Nat. Genet..

[11] Ramagopalan S.V., Dyment D.A., Cader M.Z., Morrison K.M., Disanto G., Morahan J.M. (2011). Rare variants in the CYP27B1 gene are associated with multiple sclerosis. Ann. Neurol..

